# Biomechanical Evaluation of a Novel Sutureless Hydrogel Device for Peripheral Nerve Repair

**DOI:** 10.3390/bioengineering13050551

**Published:** 2026-05-13

**Authors:** Zoe Mote, Sophia Campbell, Victoria Williams, Patryk Ostrowski, Yong Ren, Edward M. Kobraei

**Affiliations:** 1 Tulavi Therapeutics, Inc., Los Gatos, CA 95032, USA; scampbell@tulavi.com (S.C.);; 2 Department of Anatomy, Jagiellonian University Medical College, 31-008 Kraków, Poland; 3 Department of Neurosurgery/Plastic Surgery, Kaiser Permanente Redwood City/San Jose Medical Center, Redwood City, CA 94063, USA

**Keywords:** nerve repair, coaptation aid, biomaterial, sutureless repair, hand surgery, nerve conduit

## Abstract

Following traumatic injury, nerve repair is essential to the restoration of muscle function and sensation. The current gold standard of nerve repair is microsuture repair, which requires trained microsurgeons to perform time-intensive and technically demanding procedures under high magnification. Microsuture repair suffers from inconsistent repair quality among surgeons and variable clinical outcomes. Neurorrhaphy sutures are non-resorbable and prone to fibrous tissue ingrowth and attendant foreign body reaction, both of which are believed to contribute to the observed shortfall in clinical outcomes. Here, we introduce a novel, sutureless, in situ forming, and fully degradable hydrogel coaptation device for nerve repair. The practical usability of the hydrogel device was assessed by procedure timing, tensile repair strength, and repair quality compared to the traditional microsuture approach. Human cadaveric nerves were used to perform hydrogel and suture repairs for comparison in a relevant model. Additionally, the hydrogel coaptation device was used for primary sciatic nerve repairs in rats to assess feasibility for use in nerve repair in vivo. We observed that hydrogel nerve repairs were performed nearly three times faster than microsuture repairs, without any significant difference in tensile strength when pulled to failure, and had favorable quality scores when blindly assessed by plastic surgeons. Histologically, the in vivo feasibility study showed nerve bridging visualized using H&E, neurofilament, and myelin staining. Our findings suggest the novel hydrogel coaptation device may serve as a potential alternative to suture repair, with features addressing several critical limitations inherent to microsuture and existing nerve repair methods.

## 1. Introduction

Microsurgical suture neurorrhaphy remains the gold standard for peripheral nerve repair and reconstruction [1,2,3]. However, critical limitations exist with microsuture repair, including technically demanding execution, inconsistent reproducibility of repairs, iatrogenic injury to nerves, non-resorbable sutures as permanent devices, and an unfavorable impact on nerve regeneration biology [4,5,6]. Consequences of poor suture repairs can result in poor sensation, neuroma in continuity [7,8], or difficult-to-treat small fiber neuropathies [9,10]. Concurrently, the overall advancement of peripheral nerve surgery is burgeoning with new interventions and tools that are reliant on multiple nerve coaptations to achieve optimal clinical outcomes. These factors have combined to create renewed and urgent interest in alternative approaches to microsuture repair and, potentially, promise for a new “gold standard”.

Recently, a sutureless, fully resorbable, in situ forming hydrogel was developed to enable atraumatic nerve alignment, coaptation, and protection (Tulavi Therapeutics, Inc., USA) simultaneously. Comprehensive in vivo preclinical studies and human clinical experience with this hydrogel technology for a different nerve application have demonstrated biocompatibility, resorbability, and minimal inflammatory response or adhesions to surrounding tissue [11]. In this study, we compare a novel hydrogel coaptation device with traditional microsuture neurorrhaphy in a cadaver model to assess differences in biomechanical strength, time requirements, and repair quality. Finally, an in vivo feasibility study examines the device’s compatibility with nerve regeneration histologically in a sciatic nerve transection in a rat model. We hypothesized that in the cadaver study, hydrogel repairs would have biomechanical performance comparable to that of suture repairs, and that the device would support nerve regeneration histologically in vivo.

Various biomaterial coaptation aids have been previously explored in academia; however, few have been commercialized [12]. Commercially available solutions largely include tubular connector repair devices, comprising decellularized porcine small intestine submucosa (AxoGuard^®^ Nerve Connector, Axogen, Alachua, FL, USA) or collagen conduits (NeuraGen^®^ Nerve Guide, Integra, Princeton, NJ, USA). These devices need to be sutured into place, increasing potential for epineural damage and procedure time requiring microsurgical skill. Alternatively, two sutureless nerve repair devices are commercially available, one adhering to the nerve using nitinol hooks that embed in the epineurium (NerveTape^®^, BioCircuit Technologies, Atlanta, GA, USA), and another being an in situ forming polymeric device made from PGSA (COPATIUM^®^ CONNECT, TISSIUM, Paris, France). The few options available are continually improving on standard suture repair, but no consensus on a superior method has yet emerged. There exists room for improvement in usability, tissue compatibility, and atraumatic applications, which we aim to address with the device designed here. The in situ forming hydrogel, with greater than 90% water content, serves as the main support structure for nerve coaptation. This unique hydrogel device was designed to provide a soft, tissue-compatible feel and sutureless application that is distinct from existing methods, without sacrificing strength compared to sutures.

Researchers have previously explored hydrogel nerve repair devices; however; none have yet been commercialized. Many of these explorations involve growth factor delivery from the hydrogels, requiring a regulatory path of a drug–device combination product, presenting a more challenging commercial translation [13,14,15,16,17]. Here we explore a hydrogel device composed of three biomaterials with well-characterized safety and degradation mechanisms: Poly(ethylene glycol) (PEG), a tissue-native glycosaminoglycan, and poly(glycolic acid) (PGA). The precise formulations are proprietary, but the components each have undergone decades of study and use in healthcare and medical devices [18,19,20].

## 2. Materials and Methods

### 2.1. Hydrogel Coaptation Device

**Figure 1:**
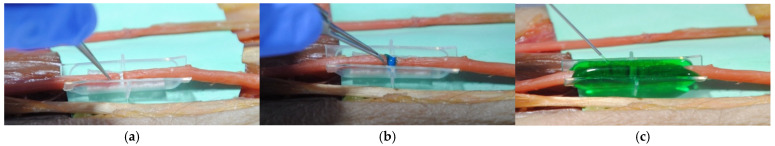
Delivery of the hydrogel coaptation device is visualized in 3 main steps. (**a**) The nerve ends are atraumatically approximated along the scaffold surface, minimizing the gap without applying tension. (**b**) A small volume of growth-permissive gel (blue) is deployed into the gap, connecting the nerve endings. (**c**) The silicone form is filled with the conformable hydrogel (green), which crosslinks rapidly, forming a soft, solid barrier around the coaptation.

The novel technology in this study is a fully sutureless and bioresorbable in situ forming hydrogel nerve coaptation device (linc™ Hydrogel Coapt, under investigation by Tulavi Therapeutics). The device comprises three elements: the aligning scaffold, growth-permissive gel (blue), and conforming hydrogel (green) (Figure 1). The scaffold is composed of poly(glycolic acid) (PGA), supports anatomic alignment of nerve endings for repair, and is permeable to the conforming hydrogel. The growth-permissive gel (GPG) is a viscous glycosaminoglycan solution that supports axonal growth and prevents the PEG gel from filling the space between nerve endings. The GPG remains as a semifluid non-crosslinked gel, but retains its shape for several minutes after delivery, allowing for precise control of placement. The conforming hydrogel is predominately composed of two uniquely formulated poly(ethylene glycol) (PEG) molecules, and when crosslinked, the resulting hydrogel is impermeable to cellular ingrowth. The PEG molecules do not require a crosslinking agent or external energy input and undergo rapid crosslinking upon mixing with a buffer solution during delivery. Crosslinking occurs within 10 s of delivery. Exact GPG and PEG formulations and combinations, as well as the aligning PGA scaffold, are patented and proprietary, and have undergone extensive characterizations for safety, biocompatibility, and biomechanical testing. All three components of the hydrogel coaptation system are fully bioresorbable by hydrolysis (PEG, PGA) and enzymatic (GPG) degradation by approximately 4 months post-implantation. Degradation components are also non-reactive and either metabolized (PGA, GPG) or cleared through the kidneys and feces (PEG) [18].

### 2.2. Application

First, a silicone form (pre-formed for nerves 1–7 mm in diameter) is selected based on the nerve diameter. The aligning scaffold is then trimmed to size and placed on the floor of the silicone form, where it provides structural support and enables precise alignment of the two nerve ends. Using micro forceps, the two nerve endings intended for coaptation are placed atraumatically into the desired alignment on the scaffold (Figure 1a).

Care is taken to ensure a less than 5 mm gap between the nerve endings. Within this gap, a small volume of GPG is deployed (Figure 1b). Next, the conforming PEG hydrogel is delivered around the coaptation site within the silicone form, penetrating the scaffold and surrounding the GPG and coaptation site to form a protective and adherent barrier (Figure 1c). Both the GPG and PEG gels are administered via syringe in liquid form. The GPG remains non-crosslinked, acting as a viscous growth substrate, and does not mix with the PEG hydrogel upon delivery. Comparatively, upon delivery as a liquid, the PEG hydrogel undergoes rapid polymerization in situ (less than 10 s) to its mature, solid hydrogel form. The coaptation site remains fully encapsulated by the hydrogel, forming both a soft (approximately 70–90 kPa compressive modulus) conduit for nerve regrowth and flexible barrier around the repair. The silicone form is then removed and discarded.

### 2.3. Cadaver Study Design

Seven fresh frozen cadaver arms from seven individuals were used in this study. Preparation of each of the specimens involved surgical exposure at three anatomic sites in each arm: proximal forearm, distal forearm, and volar hand. At the proximal and distal forearm sites, the median and ulnar nerves were dissected with a minimum of 10 cm length of exposure. At the volar hand sites, the 2nd and 3rd webspace common digital nerves were dissected with a minimum of 5 cm of exposure. No specific selection criteria were used for the specimens, which ranged in age and gender. To control for the variability, repairs for each hydrogel and suture were performed on the same specimen, e.g., one of each in the common digital nerves in the volar hand and one of each in the median and ulnar nerve in the forearm. Additionally, the regions for hydrogel vs. microsuture repair were rotated between specimens. A total of 26 nerves (14 large nerves, 12 small) were used for the study.

### 2.4. Time Comparison

Microsuture and hydrogel nerve repairs were performed in 3 cadaver specimens at all 3 sites, for a total of 18 nerve repairs (8 microsuture, 10 hydrogel). Microsuture repairs were performed by a microsurgeon under loupe magnification; hydrogel repairs were performed under direct visualization (no magnification). Each repair was timed by a single observer. Timing of microsuture repair began on grasping the first microinstrument and was completed once the microinstruments were set down following completion of the repair, which was determined at the microsurgeon’s discretion. Similarly, hydrogel repair commenced on grasping the first instrument for repair and was completed once the silicone form was removed and all instruments were set down. Hydrogel and microsuture repair durations were compared across all nerves, with additional sub-analysis performed based on nerve diameter.

### 2.5. Biomechanical Analysis

The same 18 nerve repairs (8 microsuture, 10 hydrogel) from 3 cadaveric specimens above were then assessed for linear repair strength using a Texture Analyzer (Stable Micro Systems, Godalming, UK) at a distraction rate of 40 mm/min until failure. Distraction was filmed with a high-resolution camera, with failure defined as any gapping at the repair site greater than 5 mm. For all 10 hydrogel repairs, the nerve gap containing the growth-permissive gel was less than 5 mm prior to distraction testing. Hydrogel and microsuture repair tensile strength values at failure were compared across all nerves, with additional sub-analysis based on nerve diameter.

### 2.6. Repair Quality Comparison

**Figure 2:**
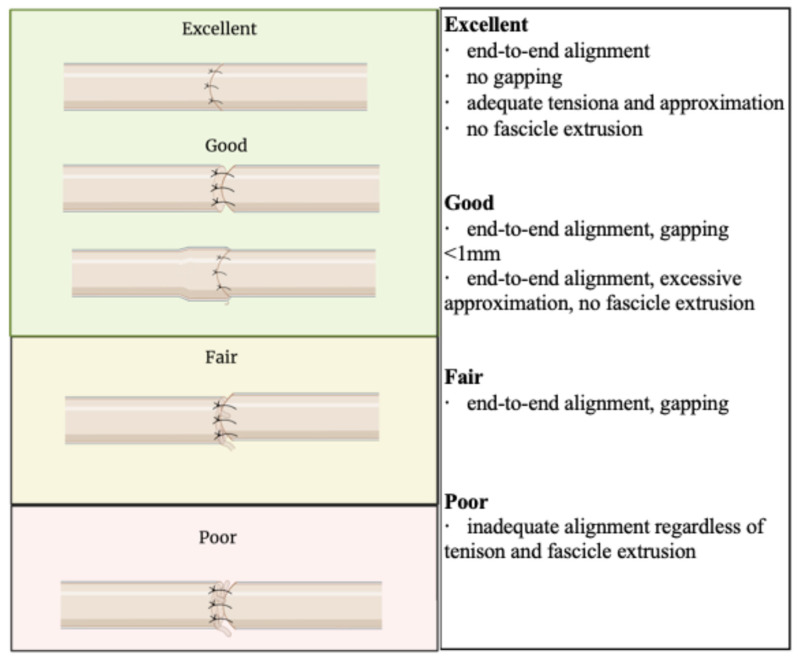
Suture repair scoring criteria adapted from [6].

**Figure 3:**
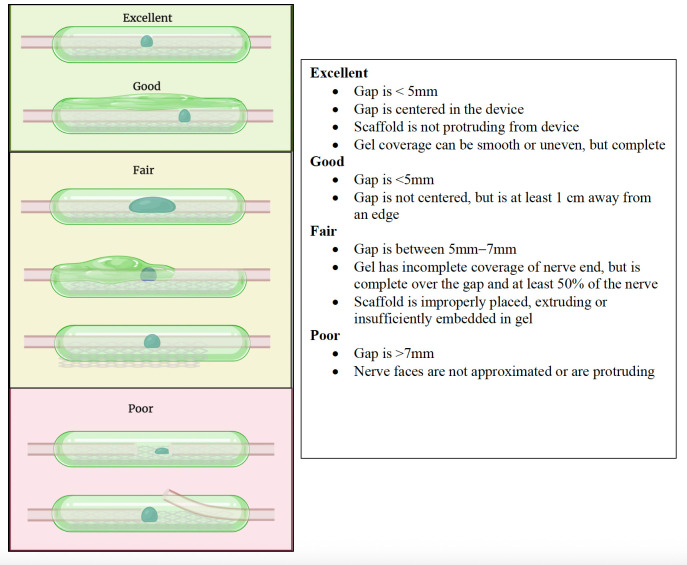
Scoring criteria for hydrogel repairs. These criteria emphasize nerve ending alignment, configuration, and gap size analogous to the suture repair criteria. Repairs that would be expected to have varying degrees of clinical success are included in fair-to-excellent categories. Only repairs that would be expected to have high likelihood of clinical failure are classified as poor to match previous suture grading scoring.

An additional 14 nerve repairs (7 microsuture, 7 hydrogel) were performed using the remaining 4 cadaver arms, and all repairs were photographed with a high-resolution camera. The same 14 nerve repairs (7 microsuture, 7 hydrogel) next underwent manual stress testing to mimic physiological stresses and immediate postsurgical joint mobilization. For the proximal and distal forearm nerve repairs, the skin was closed, and the wrist was ranged in maximum passive wrist flexion, followed by maximum passive wrist extension, for a total of 60 repetitions. For the 2nd and 3rd webspace repairs, the skin was closed, and the fingers were ranged by creating a full passively closed fist, followed by complete extension of the fingers, for a total of 60 repetitions. The skin was then opened, and the repairs photographed to permit grading analysis (Figure 2 and Figure 3). Hydrogel repair images were analyzed using Fiji ImageJ (Version 1.0.0) to measure the gap length by digital measurement between the nerve endings.

Three board-certified plastic surgeons with hand and nerve experience independently judged each repair. For microsuture repairs, a standardized nerve repair quality scale was used, with emphasis on fascicular alignment, gapping, and tension (Figure 2). An analogous grading scale was developed for hydrogel repair quality, emphasizing alignment, gapping, and degree of coverage with the conforming hydrogel (Figure 3). A cutoff gap of 5 mm was determined to standardize the length of nerve contained in the coaptations for consistency in biomechanical measurements. Additionally, 5 mm appears to be an approximation of a gap length at which many surgeons would no longer be comfortable using connectors and would instead pivot to using a graft due to risk of failed repair or neuroma in continuity [21,22,23]. Both repair quality scales were defined by the categories of Excellent, Good, Fair, and Poor, which were converted into a numerical scale (Excellent = 4; Poor = 1). Surgeon judges were presented with pre-stress test and post-stress test images in a randomized, unlabeled fashion, and were blinded to their own previously assigned scores and the scores of other judges. The Intraclass Correlation Coefficient (ICC) was calculated for scores in order to assess interrater reliability [24].

### 2.7. In Vivo Feasibility Study

A preclinical pilot study evaluated the ability of the sutureless hydrogel device to support nerve regeneration in an established rat model of sciatic-nerve transection.

Nerve regeneration in vivo was assessed grossly through histopathological evaluation at 6 weeks post-surgery.

#### 2.7.1. Surgical Procedure

Five (5) Lewis rats underwent left sciatic nerve transection and repair using the hydrogel coaptation device (Tulavi Therapeutics, Los Gatos, CA, USA). Pre-operatively, animals were dosed with 0.65 mg/kg Ethiqa XR for pain management. Rats were induced under 4% isoflurane and maintained at 2% for pre-operative care and surgery. Pre-operatively, rats were shaved and cleaned using successive iodine and 70% isopropyl alcohol wipes and provided ear tags and eye lubricant. Surgically, the left sciatic nerve was exposed using a standard gluteal-muscle splitting incision. Approximately 2 cm of nerve was exposed and freed from connective tissue to support mobility proximal to the trifurcation. The nerve was transected using micro scissors, with no nerve tissue removed. The hydrogel was applied using the technique described above using a custom rat-sized silicone form for nerves approximately 1 mm in diameter. The gap between nerve endings was measured using a surgical ruler, ensuring they were consistently 1–2 mm for all animals. The muscle and skin were closed using 4-0 degradable suture, and animals were treated with 1 mg/kg Meloxicam subcutaneously for post-operative pain immediately and for 2 days after surgery. Animals were allowed to survive until 6 weeks, then humanely euthanized. At necropsy, sciatic nerves were excised and fixed in 4% paraformaldehyde (VWR) before being transferred to 70% Isopropyl alcohol for histopathology.

#### 2.7.2. Histopathology

Nerve samples underwent paraffin embedding, sectioning, staining, and analysis by an expert pathology facility (StageBio, Frederick, MD, USA). Nerve sections were stained with Hematoxylin and Eosin (H&E) as well as immunostains for Neurofilament-Heavy Chain (NF-H) and Myelin Basic Protein (MBP). Sections were analyzed by a board-certified pathologist for conclusions regarding nerve healing and immune response.

### 2.8. Statistical Analysis

All statistical analyses were performed using statistical software (GraphPad Prism version 11.0.0, GraphPad Software, LLC, Boston, MA, USA). Biomechanical data and time assessment were assessed with an unpaired two-tailed *t*-test between the groups, with *p* < 0.05 considered statistically significant. Linear regression was used to assess any correlation of either suture number (for microsuture repairs) or nerve diameter (for hydrogel repairs) with tensile strength. Descriptive statistics and Coefficients of Variation were calculated within groups for quality measurements. For interclass correlation coefficients (ICC), an ANOVA was performed for all quality scores by a scorer to generate coefficients for ICC calculation. The means squares within and between were used to calculate the ICC [24]. In text, data are represented as means ± standard deviation.

## 3. Results

### 3.1. Time Comparison

**Figure 4:**
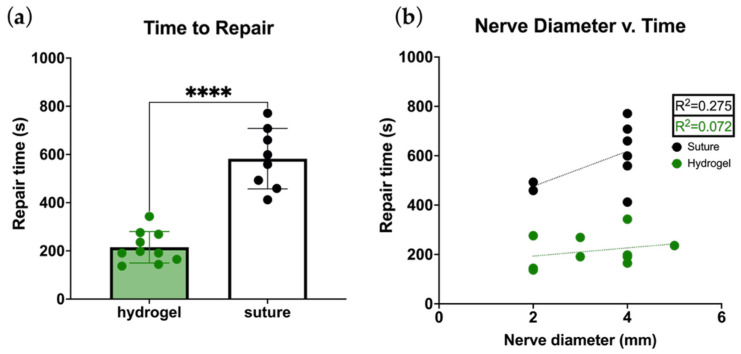
(**a**) Hydrogel repairs are significantly faster to perform than suture repairs. Data represented as mean ± SD, **** indicates *p* < 0.0001 with unpaired two tailed *t*-test. (**b**) Hydrogel repair duration was similar across all nerve diameters, but microsuture repair appears to have increased time requirements for larger nerves. Trendlines are shown in dotten lines.

Hydrogel repairs were significantly faster than microsuture repairs, requiring an average 3:35 ± 1:05 min compared to 9:43 ± 2:05 min, respectively (Figure 4a). Increasing nerve diameter may correlate with increasing duration for microsuture repairs, but not with the duration of hydrogel repairs (Figure 4b).

### 3.2. Biomechanical Analysis

**Figure 5:**
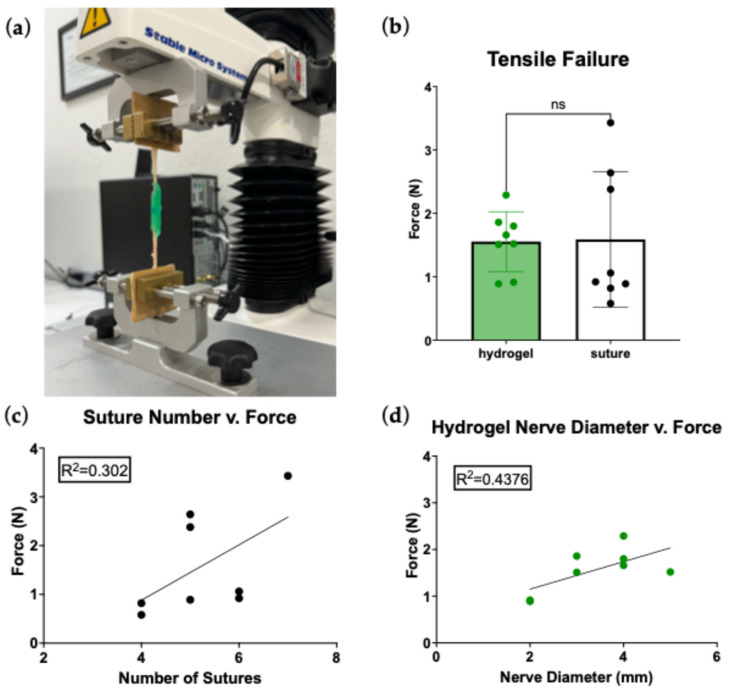
(**a**) Representative image of tensile testing on Texture Analyzer; (**b**) the force at which the repair methods failed was measured. Data represented as mean ± SD, no significant difference (*p* = 0.9347); (**c**) for suture repairs, the strength is potentially correlated to the number of sutures in the repair; (**d**) for hydrogel repairs, the strength may be correlated with nerve diameter.

Hydrogel and microsuture repairs demonstrated no statistical difference in tensile strength of repair (*p* = 0.9347, Figure 5b). Hydrogel nerve repairs all failed through a mode of hydrogel detachment from the epineurium at an average of 1.56 ± 0.47 N, while suture repairs failed either through suture rupture or epineural tear at an average of 1.59 ± 1.07 N. To further understand the mechanism of repair strength for each method, potential correlations were investigated. While limited by the number of samples, both repair types appeared to exhibit a potential correlation with nerve size and repair tensile strength. In microsuture repairs, the number of sutures may correlate with repair strength (Figure 5c); however, in hydrogel repairs, nerve diameter may correlate directly with greater repair strength (Figure 5d).

### 3.3. Repair Quality Comparison

**Figure 6:**
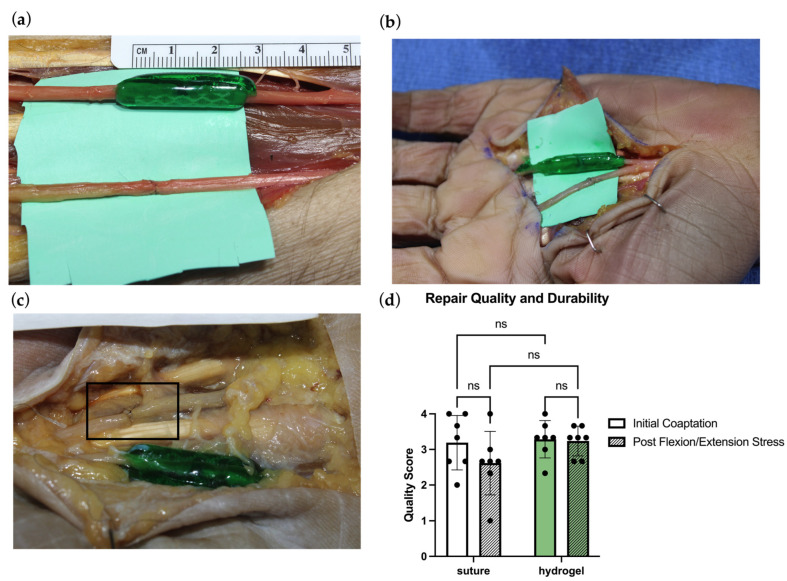
(**a**) Representative suture and hydrogel repairs in a cadaver forearm. Both suture and hydrogel repairs in this image received a score of 3.33. The gap length in this hydrogel repair was measured to be 1.346 mm. (**b**) Representative suture and hydrogel repairs in the 2nd and 3rd webspace of the hand. (**c**) Dehiscence of a microsuture repair after manual stress testing with 60 repetitions of flexion and extensions is shown in the black box. Note the preserved adjacent hydrogel nerve repair after the same stresses. (**d**) Repair quality scores at the initial coaptation compared to the quality scores after the repairs underwent 60 flexion and extension repetitions.

All hydrogel nerve coaptations had an average gap length of 1.61 ± 1.19 mm, with the largest gap at 4 mm (Table 1). After manual flexion/extension stress testing of the repairs, the average gap length did not undergo a statistically significant change (*p* = 0.8158), with an average post-stress testing gap of 1.78 ± 1.35 mm. Favorable repair quality was observed with initial hydrogel and microsuture repairs, with both repair types achieving “good” to “excellent” average scores in their respective scales (average microsuture repair score: 3.19 ± 0.77; the average hydrogel repair score: 3.29 ± 0.52) (Figure 6d). After manual stress-testing of the repairs with flexion/extension of the wrists and digits, no noteworthy change in repair quality was identified. However, the microsuture repair group showed a trend toward reduction in repair quality that was not seen in the hydrogel repair group (2.619 ± 0.89; 3.238 ± 0.42). One microsuture repair experienced a dehiscence following manual stress testing and was categorized as “poor” (a score of 1) by all raters (Figure 6c). Interrater reliability was assessed using the ICC measured in each scoring system. The ICC was calculated to be 0.61 for suture repair scoring, and only 0.47 for hydrogel, indicating moderate and low alignment between raters, respectively (0–1). However, the Coefficients of Variation for microsuture repair quality both before and after stress (24.025% CV and 34.016% CV) were much greater than those observed for hydrogel repairs (15.967% CV and 12.910% CV) (Table 2).

### 3.4. In Vivo Performance

**Figure 7:**
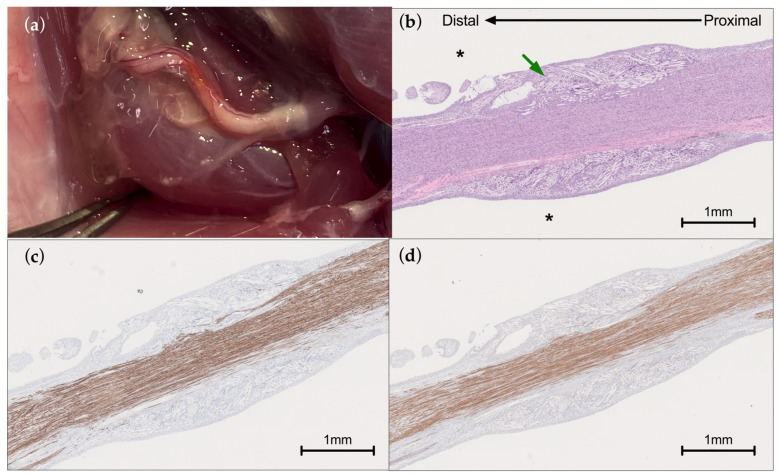
(**a**) Gross image of nerve repaired with hydrogel prior to explant at 6 weeks. Blood vessels are visible through remaining transparent gel material. (**b**) Hematoxylin and Eosin staining. (**c**) Neurofilament-heavy chain staining and (**d**) myelin basic protein staining across the center of the device. Gaps of 1–2 mm in all animals were previously centered in the device and are no longer visible. White fibers identified by the green arrow are partially degraded PGA scaffold fibers. PEG hydrogel was present in the external void space (*) but dissolved during staining.

At 6 weeks, the histology demonstrated sciatic nerve regeneration across the gap (Figure 7). Figure 7a shows grossly a nerve treated with the hydrogel at 6 weeks prior to explanting for histology. Partially degraded gel is visible through the center of the pictured region. Blood vessels and nerve tissue are also visible through the transparent gel material. The reviewing pathologist noted a lack of inflammation visible in the H&E images for all five animals, which can be seen by the lack of infiltrating immune cells and normal morphology in Figure 7b. Staining for neurofilament heavy-chain shows regenerated axons bridging across the previous gap region in the center of the device (Figure 7c). Finally, myelin basic protein staining demonstrates that axons regenerating across the gap are also remyelinating (Figure 7d). Results were consistent across all five animals.

## 4. Discussion

Peripheral nerve surgery is advancing at a remarkable pace; however, surgeons face fundamental and ongoing unsolved challenges inherent to surgical practice that result in suboptimal clinical outcomes following treatment to restore critical functions. Methods to improve consistency, speed, and outcomes are increasingly necessary given the growing reliance on nerve coaptations in procedures ranging from nerve transfers, targeted muscle reinnervation, and nerve grafting to primary nerve repairs. Here, we demonstrate the practical usability of the hydrogel coaptation device compared to traditional microsuture repair as well as feasibility with in vivo preclinical performance.

The hydrogel coaptation device was designed to specifically address the inherent shortcomings of existing repair methods. Current nerve repair technologies attempt to enhance one or more of the following functions of nerve repair: coaptation function, repair strength function, channel function, and barrier function.

### 4.1. Coaptation Function

Historic and present-day nerve coaptation is built on a foundation of suture and later microsuture repair, which has been the gold standard. However, five decades of studies on suture neurorrhaphy have unveiled critical limitations. Suture materials such as catgut, polyglycolic acid, polypropylene, and monofilament nylon induce a robust inflammatory response, characterized by migration of fibroblasts and collagen deposition at the suture line [25,26,27,28]. This increased inflammation and collagen deposition translate into smaller compound action potentials, slower conduction velocities, and a reduction in axon fiber counts crossing the repair sites in animal models [29]. Animal studies have even shown a dose-dependent deleterious effect of the number of sutures on both electrophysiological outcomes and collagen deposition in the epineurium when three-suture neurorrhaphy is compared to six-suture neurorrhaphy [4]. Additionally, the practice of microsuture repair is time-consuming, technically demanding, and not readily reproducible across different surgeons and nerves [5,6]. Collectively, these factors contribute to the inconsistent and overall poor clinical outcomes observed [30].

In this inaugural study of hydrogel coaptation, application was rapid, taking less than a third of the time required for microsuture repair, and time for repair was not affected by nerve size. Notably, microsuture repairs were performed using loupe magnification rather than a microscope, likely slowing the repair time measurably. However, in addition to slower times, microsuture repair duration showed a clear dependence on nerve size (Figure 4b), an important consideration when increasing suture numbers are both deleterious and-time intensive, but sufficient suture volume is required for repair strength.

### 4.2. Repair Strength Function of Nerve Repair

The repair strength function characterizes the nerve repair’s ability to resist forces causing gapping and dehiscence for the first approximately 3–4 weeks during which the nerve repair site remains structurally vulnerable [31,32]. No difference in repair strength was observed between microsuture and hydrogel nerve repairs (Figure 5). Not surprisingly, a correlation is likely between number of sutures and repair strength for microsuture repairs, but with substantial added time for larger nerve repairs. For hydrogel repairs, repair strength increased with increasing nerve diameter; however, even small nerve hydrogel repairs were comparable in strength to microsuture repairs. The increased repair strength of hydrogel coaptations for larger-diameter nerves likely reflects a surface area-dependent adhesion mechanism. The PEG hydrogel is delivered as a liquid, where it interdigitates around the micro surface architecture of the epineurium before crosslinking in place. This increased surface contact area translates into increased resistance to gapping forces. Additionally, when exposed to physiologically relevant tensions during full flexion and extension of the relevant joints, no visible reduction in quality was identified, indicating the tensile strength provided by the hydrogel is sufficient for relevant movement in the anatomical region explored. Other materials have been investigated for their potential to achieve simultaneous coaptation and repair strength functions without the use of sutures. Fibrin glue has been used extensively for this purpose; however, it has a far weaker repair strength than sutures and is unsuitable for resistance to mechanical stresses, with dehiscence reported in 6–80% of repairs [33,34]. Still, its favorable biocompatibility, technical simplicity, and sutureless application support its wide use in nerve repair.

Recently, a medical device based on an animal xenograft biologic substrate with integrated nitinol hooks (NerveTape™, Biocircuit Technologies) has reported repair strength superior to that of sutures in nerve coaptation. Cadaveric and early clinical experience suggest the device has equal or greater tensile strength than microsuture repairs, reduced skill dependence, reduced time required for repair, and significantly improved repair quality compared to microsuture repair alone [35,36,37]. However, the device fundamentally relies on a traumatic application, where sharp nitinol hooks directly penetrate the epineurium along a significant length of both nerve termini starting near the coaptation site. While this may be suitable for repairs that are expected to undergo a significant degree of tension, the potential risks of nitinol hooks may exceed that of permanent sutures. Early results compared to microsuture show no difference in clinical outcomes with the device [38,39,40,41]. In that light, the hydrogel repair represents an alternative based on a completely atraumatic application and lack of permanent materials. The hydrogel device presented here can be easily removed, redelivered, and undergo revisions, unlike any attempt to remove, adjust, or replace metal hooks or sutures from the nerve. Other sutureless approaches build on experience with synthetic glues, polymer sealants, and laser or light-assisted photothermal bonding [42,43]. The relative value, efficacy, and pragmatism of these approaches remain to be determined. Preclinical work has explored various other hydrogel and biomaterial conduits for nerve repair over the years, yet few have evolved to full clinical translation.

### 4.3. Channel Function of Nerve Repair

Broadly, commercial nerve repair technologies function by forming a conduit or channel through which nerve regeneration occurs. The channel function of nerve repair is dependent on the internal environment of the nerve repair interface itself; the size of the gap, the alignment of nerve endings, and the presence or absence of tension, fascicular extrusion, or buckling. The channel function concept is the basis for repair quality grading scales and summarizes the three-dimensional configuration in which axons are guided toward their targets (Figure 2 and Figure 3).

Connector-assisted repairs (CAR) were popularized to overcome limitations around the technical difficulty and inconsistent quality of microsuture repairs, while also avoiding microsuture placement directly at the coaptation site. Commercially available connectors typically consist of collagen or porcine small intestine submucosa (SIS) xenografts formed into a cylindrical tube to contain the coaptation site. Several studies have since evaluated CAR, suggesting equivalent or better clinical outcomes compared to standard microsuture repair [38,39,40]. Recent data suggest CAR improves biology at the coaptation site with improved vascularity and axon counts and reduced, albeit still present, inflammatory response when compared to suture repairs alone [44]. Despite tangible benefits, CAR affords limited control over the terminal nerve endings, and the associated gap size does not obviate microsuture placement or the use of sutures; however, repair quality is still linked with surgeon experience [6]. The materials used for these connectors are often animal-derived and remodeled into tissues rather than cleared from the body.

Hydrogel nerve coaptation was designed to address each of these key limitations. The hydrogel delivery method utilizes the growth-permissive gel both as a growth substrate and as a channel-forming material for regenerating axons. The surrounding PEG hydrogel is impermeable to cells and blocks axonal escape from the repair site, further enhancing the channel function. In our repair quality evaluation, initial repair quality was rated in the good–excellent range for both microsuture and hydrogel repair groups; however, after manual stress testing, hydrogel repairs largely preserved their quality scores, while microsuture repair quality declined (Figure 6d). One dehiscence occurred following manual stress testing, involving microsuture repair of a 3rd webspace common digital nerve, while the adjacent 2nd webspace common digital nerve hydrogel coaptation remained unaffected by the same manual stresses (Figure 6c). Hydrogel coaptations also exhibited less variation in repair quality scores compared to microsuture repairs, both before and after manual stress testing (Table 2). These findings exemplify the elasticity of the hydrogel when subject to non-failure deformation, conferring elastic properties which may support earlier mobilization of joints following hydrogel nerve repair compared to existing coaptation methods.

Hydrogel coaptation also permitted precise positioning of both nerve endings with minimal forceps manipulation, avoiding fascicular damage or buckling, rotation, or misalignment that can occur with microsuture repair or CAR. The desired configuration at the coaptation site is easily and precisely set before the hydrogel preserves the entire construct once the PEG hydrogel polymerizes and solidifies. The ease of application (no prior training, no sutures, no loupe magnification required) highlights the potential for hydrogel nerve repairs to permit reproducible, high-quality nerve coaptations across a broad range of surgical expertise and experience.

### 4.4. Barrier Function of Nerve Repair

The barrier function relates to the interchange of cells, macromolecules, signaling cues, and fluids between the internal environment of the coaptation site and the external local tissue environment. Ideal barrier materials shield the coaptation site from injurious substances, such as inflammatory signaling and fibroblast proliferation and migration. Simultaneously, such materials would concentrate local signaling cues, encourage angiogenesis, and support endothelial and Schwann cell migration within the regenerative interface. Additionally, an effective barrier material would be entirely bioresorbable following the timeframe of nerve regeneration while eliciting minimal inflammatory response that could result in adhesions and fibrosis. Unfortunately, such an ideal barrier biomaterial is not currently available for clinical use.

Microsuture repair alone results in poor barrier function, with an incomplete and non-selective influx of inflammatory molecules as well as efflux of critical intraneural fluids and local guidance cues [5]. There is recent clinical interest in influencing barrier function using increasingly available nerve-protective “wraps” built on porcine SIS, type I collagen, chitosan, and human amniotic membrane among other materials [33]. These wraps are often used to enhance suture coaptations, but data supporting efficacy in nerve regeneration are inconclusive [45,46].

Recently, a sutureless conduit formed from poly(glycerol co-sebacate) acrylate (PGSA) has been cleared for nerve repair (COAPTIUM CONNECT^®^, Tissium, Paris, France). This device consists of a preformed PGSA tube that is secured to the nerve using a light-curable form of PGSA. This device forms a channel and barrier with a robust but degradable polymer material. Biomechanical repair strength data also showed no significant difference from suture repair [47]. Preclinical results with the device in a sciatic nerve repair model have shown no difference from suture repairs in nerve conduction or axon density at 3 months [48]. This device represents a shift towards atraumatic delivery and biodegradability, while maintaining results similar to those of suture repair. Materially, PGSA is biodegradable over a longer period (undefined), and the stiffnesses have been reported as 2.5 MPa and 18.2 MPA for the two components [49]. In contrast, the PEG formulation in the hydrogel coaptation device studied here has a stiffness similar to nerve tissue (12–54 kPa) at 70–90 kPa, more than an order of magnitude softer than the PGSA device [50].

Another crucial element of the barrier function is preventing adhesions and scar tissue formation between the repair site and the surrounding tissues. As demonstrated in the in vivo analysis (Figure 7), there is unrestricted axonal bridging across the gap, with no visible scar deposition around the repair site. By 6 weeks, the hydrogel is partially degraded, with very soft gel remaining in a layer outside of the nerve. Histologically, this remaining PEG hydrogel component is not visible and appears as the void space around the tissue due to the lack of infiltrating cellular or extracellular matrix components. The PGA scaffold degrades more slowly and can be seen still surrounding the nerve. Blood vessels are visible though the transparent gel grossly (Figure 7a). The lack of infiltrating immune cells in the healing nerve was noted in all five samples by the reviewing pathologist, indicating low immune reactivity to the device materials at this time point [51]. Clinical examination of a commercial hydrogel (allay™ Nerve Cap, Tulavi Therapeutics) built on the same PEG hydrogel technology demonstrated a complete absence of inflammatory response and adhesions to surrounding tissue types (bone, tendon, muscle) at 3 months post implantation in a patient undergoing conversion of below-knee amputation to above-knee amputation [11]. The hydrogel‘s minimal inflammatory response is perhaps related to its non-reactive components and biological degradation, which is mediated through hydrolysis to bioinert degradants that are cleared by the liver and kidneys [52,53].

There are several important limitations to this study. For biomechanical and quality analysis, cadaveric nerves were used. Fresh, non-fixed cadavers were selected to minimize differences from living nerves and allow for physiologically relevant stress testing; cadaver nerves do not perfectly replicate living human nerve tissue. Our small sample size limited statistical performance and may require confirmatory work with higher statistical power; however, it did not prevent demonstration of the clear trends that were observed. All microsuture repairs were performed by a single microsurgeon under loupe magnification, whereas, in practice, an operating microscope is often used, which could have important implications for suture repair quality and speed. The scales for repair quality assessments were also different between the groups, but categories were assigned so that each category had similar levels of risk based on clinical experience, and statistical comparisons between the groups we not made. Given the known bioresorbable nature of the hydrogel coaptation system, this study did not evaluate the variation in repair strength over time with hydrogel repairs. Finally, the in vivo histology presented only served as a feasibility study, and follow-up with well-powered animal studies with comparator arms and functional outcomes is required to corroborate or refute the findings observed in this study.

## 5. Conclusions

Hydrogel coaptation was much faster than microsuture repairs and did not differ based on the size of nerve. Both hydrogel and microsuture repairs yielded comparable repair tensile strength and had favorable repair quality scores; however, hydrogel repairs exhibited less variability and better preservation of repair quality after manual stress testing. Technical application was facile, atraumatic, and precise. In vivo, the hydrogel coaptation supported nerve regeneration across the gap with no signs of excess inflammation or scarring. The results of this study, coupled with the uniquely favorable physical and biological properties of the hydrogel coaptation system, show its promise as a potential new strategy in nerve repair.

## 6. Patents

U.S. Patent No. 11944717 has been granted for the technology described here. Other patents pending.

## Figures and Tables

**Table 1:** Gap length of hydrogel repairs was digitally measured and recorded prior to quality scoring.

Specimen Number	Region	Nerve	Initial Gap Length (mm)	Post-Stress Testing Gap Length (mm)
Specimen 1	Hand	Common digital 3rd webspace	0.85	0.89
Specimen 2	Hand	Common digital 2nd webspace	4.04	4.60
Specimen 3	Hand	Common digital 3rd webspace	1.09	1.24
Specimen 4	Hand	Common digital 2nd webspace	1.03	1.33
Specimen 1	Forearm	Ulnar	1.35	1.37
Specimen 2	Forearm	Median	2.25	2.3
Specimen 3	Forearm	Ulnar	0.68	0.69
		Mean ± SD	1.61 ± 1.19 mm	1.78 ± 1.35 mm

**Table 2:** Mean score variability between repair types, represented by coefficients of variation.

	Initial Repair			Post-Flexion/Extension Stress		
	Mean Score	Coefficient of Variation (% CV)	n	Mean Score	Coefficient of Variation (% CV)	n
**Suture**	3.190	24.025	7	2.619	34.016	7
**Hydrogel**	3.286	15.967	7	3.238	12.910	7

## Data Availability

The data presented in this study are not available on request from the corresponding author due to ongoing regulatory work.

## References

[1] Habre S.B., Bond G., Jing X.L., Kostopoulos E., Wallace R.D., Konofaos P. (2018). The Surgical Management of Nerve Gaps: Present and Future. Ann. Plast. Surg..

[2] Siemionow M., Brzezicki G. (2009). Current Techniques and Concepts in Peripheral Nerve Repair. Int. Rev. Neurobiol..

[3] Pereira C., Hill E., Stasyuk A., Parikh N., Dhillon J., Wang A., Li A. (2023). Molecular Basis of Surgical Coaptation Techniques in Peripheral Nerve Injuries. J. Clin. Med..

[4] Martins R.S., Teodoro W.R., Simplício H., Capellozi V.L., Siqueira M.G., Yoshinari N.H., Plese J.P.P., Teixeira M.J. (2011). Influence of Suture on Peripheral Nerve Regeneration and Collagen Production at the Site of Neurorrhaphy: An Experimental Study. Neurosurgery.

[5] Barton M.J., Morley J.W., Stoodley M.A., Lauto A., Mahns D.A. (2014). Nerve Repair: Toward a Sutureless Approach. Neurosurg. Rev..

[6] Isaacs J., Safa B., Evans P.J., Greenberg J. (2016). Technical Assessment of Connector-Assisted Nerve Repair. J. Hand Surg..

[7] Walsh A.R., Beutel B.G., Tordjman D., da Costa A.C., Melamed E. (2023). Neuroma-in-continuity: A review of pathophysiology and approach to the affected patient. Hand Surg. Rehabil..

[8] Vesterholm K., Troest R.W., Gvozdenovic R. (2025). Challenges in the surgical treatment of neuroma in continuity in the upper extremity using human acellular nerve allografts. J. Plast. Reconstr. Aesthetic Surg..

[9] Levinson J., Collins R., Singla P. (2024). A Review of Small Fiber Neuropathy. ASRA News.

[10] Vecchio M., Chiaramonte R., Romano M., Pavone P., Musumeci G., Mauro G.L. (2020). A systematic review of pharmacologic and rehabilitative treatment of small fiber neuropathies. Diagnostics.

[11] Ostrowski P., Deng I., Kobraei E.M. (2025). A Novel Hydrogel for Treatment and Prevention of Symptomatic Neuroma: Early Clinical Experience. Plast. Reconstr. Surg. Glob. Open.

[12] Belkas J.S., Munro C.A., Shoichet M.S., Midha R. (2005). Peripheral nerve regeneration through a synthetic hydrogel nerve tube. Restor. Neurol. Neurosci..

[13] Midha R., Munro C.A., Dalton P.D., Tator C.H., Shoichet M.S. (2003). Growth factor enhancement of peripheral nerve regeneration through a novel synthetic hydrogel tube. J. Neurosurg..

[14] Taisescu O., Dinescu V.C., Rotaru-Zavaleanu A.D., Gresita A., Hadjiargyrou M. (2025). Hydrogels for Peripheral Nerve Repair: Emerging Materials and Therapeutic Applications. Gels.

[15] Gnavi S., di Blasio L., Tonda-Turo C., Mancardi A., Primo L., Ciardelli G., Gambarotta G., Geuna S., Perroteau I. (2017). Gelatin-based hydrogel for vascular endothelial growth factor release in peripheral nerve tissue engineering. J. Tissue Eng. Regen. Med..

[16] Xu H., Yu Y., Zhang L., Zheng F., Yin Y., Gao Y., Li K., Xu J., Wen J., Chen H. (2022). Sustainable release of nerve growth factor for peripheral nerve regeneration using nerve conduits laden with Bioconjugated hyaluronic acid-chitosan hydrogel. Compos. B Eng..

[17] Jhaveri S.J., Hynd M.R., Dowell-Mesfin N., Turner J.N., Shain W., Ober C.K. (2009). Release of nerve growth factor from HEMA hydrogel-coated substrates and its effect on the differentiation of neural cells. Biomacromolecules.

[18] Webster R., Elliott V., Park B.K., Walker D., Hankin M., Taupin P. (2009). PEG and PEG Conjugates Toxicity: Towards an Understanding of the Toxicity of PEG and Its Relevance to PEGylated Biologicals.

[19] D’souza A.A., Shegokar R. (2016). Polyethylene glycol (PEG): A versatile polymer for pharmaceutical applications. Expert Opin. Drug Deliv..

[20] Jang H.J., Shin C.Y., Kim K.B. (2015). Safety evaluation of polyethylene glycol (PEG) compounds for cosmetic use. Toxicol. Res..

[21] Isaacs J., Browne T. (2014). Overcoming short gaps in peripheral nerve repair: Conduits and human acellular nerve allograft. Hand.

[22] Tan R.E.S., Jeyaratnam S., Lim A.Y.T. (2023). Updates in peripheral nerve surgery of the upper extremity: Diagnosis and treatment options. Ann. Transl. Med..

[23] Lundborg G., Rosén B., Dahlin L., Holmberg J., Rosén I. (2004). Tubular repair of the median or ulnar nerve in the human forearm: A 5-Year follow-up. J. Hand Surg..

[24] Koo T.K., Li M.Y. (2016). A Guideline of Selecting and Reporting Intraclass Correlation Coefficients for Reliability Research. J. Chiropr. Med..

[25] DeLee J.C., Smith M.T., Green D.P. (1977). The reaction of nerve tissue to various suture materials: A study in rabbits. J. Hand Surg. Am..

[26] Bratton B.R., Kline D.G., Hudson A.R., Coleman W.T. (1981). Use of monofilament polyglycolic acid suture for experimental peripheral nerve repair. J. Surg. Res..

[27] Cham R.B., Peimer C.A., Howard C.S., Walsh W.P., Eckert B.S. (1984). Absorbable versus nonabsorbable suture for microneurorrhaphy. J. Hand Surg. Am..

[28] Hudson A.R., Hunter D. (1976). Polyglycolic Acid Suture in Peripheral Nerve II: Sutured Sciatic Nerve. Can. J. Neurol. Sci./J. Can. Des. Sci. Neurol..

[29] Atkins S., Smith K.G., Loescher A.R., Boissonade F.M., O’Kane S., Ferguson M.W., Robinson P.P. (2006). Scarring Impedes Regeneration at Sites of Peripheral Nerve Repair. Neuroreport.

[30] Ruijs A.C.J., Jaquet J.-B., Kalmijn S., Giele H., Hovius S.E.R. (2005). Median and Ulnar Nerve Injuries: A Meta-analysis of Predictors of Motor and Sensory Recovery After Modern Microsurgical Nerve Repair. Plast. Reconstr. Surg..

[31] Abrams R.A., Butler J.M., Bodine-Fowler S., Botte M.J. (1998). The Tensile Properties of the Neurorrhaphy Site in the Rat Sciatic Nerve. J. Hand Surg..

[32] Higgs P.E., Weeks P.M. (1979). The Rate of Bursting Strength Gain in Repaired Nerves. Ann. Plast. Surg..

[33] Clifford A.L., Klifto C.S., Li N.Y. (2024). Nerve Coaptation in 2023: Adjuncts to Nerve Repair Beyond Suture. J. Hand Surg. Glob. Online.

[34] Koopman J.E., Duraku L.S., de Jong T., de Vries R.B., Zuidam J.M., Hundepool C.A. (2022). A Systematic Review and Meta-analysis on the Use of Fibrin Glue in Peripheral Nerve Repair: Can We Just Glue It?. J. Plast. Reconstr. Aesthetic Surg..

[35] Eberlin K.R., Safa B., Buntic R., Rekant M.S., Richard M.J., Styron J.F., Bendale G., Isaacs J. (2024). Usability of Nerve Tape: A Novel Sutureless Nerve Coaptation Device. J. Hand Surg. Am..

[36] Bendale G.S., Sonntag M., Clements I.P., Isaacs J.E. (2022). Biomechanical Testing of a Novel Device for Sutureless Nerve Repair. Tissue Eng. Part C Methods.

[37] Chinta M., Webster T., Huang H., Gfrerer L. (2025). Sense and Sensibility: Nerve Tape—A More Efficient Alternative for Nerve Repair in Breast Reconstruction?. Plast. Reconstr. Surg. Glob. Open.

[38] Ducic I., Safa B., DeVinney E. (2017). Refinements of nerve repair with connector-assisted coaptation. Microsurgery.

[39] Agosti E., Zeppieri M., Ius T., Antonietti S., Gelmini L., Denaro L., Bonetti A., Fontanella M.M., Ortolani F., Panciani P.P. (2025). Comparative Outcomes of Direct Versus Connector-Assisted Peripheral Nerve Repair. Biomedicines.

[40] Leis A., Smetana B.S., Strohl A.B., Styron J.F. (2024). Comparative Effectiveness Systematic Review and Meta-analysis of Peripheral Nerve Repair Using Direct Repair and Connector-assisted Repair. Plast. Reconstr. Surg. Glob. Open.

[41] Bendale G.S., Drinane J., Savsani K., Phan K., Joshi R., Isaacs J. (2025). Nerve Tape Is an Effective Tool for Small Nerve Repair. Plast. Reconstr. Surg. Glob. Open.

[42] Huang T.C., Blanks R.H.I., Berns M.W., Crumley R.L. (1992). Laser VS. Suture Nerve Anastomosis. Otolaryngol.–Head Neck Surg..

[43] Soucy J., Sani E.S., Portillo-Lara R., Diaz D., Dias F., Weiss A.S., Koppes A., Koppes R.A., Annabi N. (2018). Photocrosslinkable Gelatin/Tropoelastin Hydrogel Adhesives for Peripheral Nerve Repair. Tissue Eng. Part A.

[44] Alsmadi N.Z., Deister C., Evans P., Ghanem T., Smetana B., Mercer D. (2025). Protecting the Nerve Coaptation: Connector-Assisted Nerve Repair in Complex Injuries. J. Hand Surg. Glob. Online.

[45] Zhu X., Wei H., Zhu H. (2018). Nerve wrap after end-to-end and tension-free neurorrhaphy attenuates neuropathic pain: A prospective study based on cohorts of digit replantation. Sci. Rep..

[46] Thomson S.E., Ng N.Y., O Riehle M., Kingham P.J., Dahlin L.B., Wiberg M., Hart A.M. (2022). Bioengineered Nerve Conduits and Wraps for Peripheral Nerve Repair of the Upper Limb. Cochrane Database of Systematic Reviews.

[47] Wlodarczyk A.I., Collin E.C., Pereira M.J., Bindra R.M., Power D.M.M. (2024). Biomechanical Evaluation of an Atraumatic Polymer-Assisted Peripheral Nerve Repair System Compared with Conventional Neurorrhaphy Techniques. Plast. Reconstr. Surg. Glob. Open.

[48] Nguyen D., Collin E., de Miguel R., Reyes-Gomez E., Shah A., Eberlin K.R. (2025). A Novel Atraumatic Polymer-Assisted Peripheral Nerve Repair Device Compared with Microsurgical Neurorrhaphy. J. Hand Surg. Glob. Online.

[49] Xylas J., Collin E., Menand S., Maia J., Pereira M. (2022). Sutureless Nerve Coaptation Leveraging a Novel Bioinspired Light-Activated Polymer Platform: Poly(glycerol-co-sebacate) Acrylate (PGSA).

[50] Ma Z., Hu S., Tan J.S., Myer C., Njus N.M., Xia Z. (2013). In vitro and in vivo mechanical properties of human ulnar and median nerves. J. Biomed. Mater. Res. A.

[51] Choudhary S., Dubey A., Singh A., Zamboni P., Gupta N., Singh R., Tisato V., Aggarwal L.M., Gemmati D. (2025). Engineering the microenvironment: Advanced biomaterials for humanized in vitro immunotoxicology and carcinogenicity assessment. Explor. BioMat-X.

[52] Yamaoka T., Tabata Y., Ikada Y. (1994). Distribution and Tissue Uptake of Poly(Ethylene Glycol) with Different Molecular Weights after Intravenous Administration to Mice. J. Pharm. Sci..

[53] Yamaoka T., Tabata Y., Ikada Y. (1995). Fate of Water-Soluble Polymers Administered via Different Routes. J. Pharm. Sci..

